# “Mirrored” Rolando's Fracture of the Base of the Fifth Metacarpal

**Published:** 2014-10-27

**Authors:** Saptarshi Biswas, Rushyuan Lee, Arpit Patel, Scott Lifchez

**Affiliations:** Johns Hopkins University Medical Center, Baltimore, Md

**Keywords:** intra-articular fracture, fifth metacarpal, Bennett's fracture, Rolando's fracture, mirrored Rolando

**Figure:**
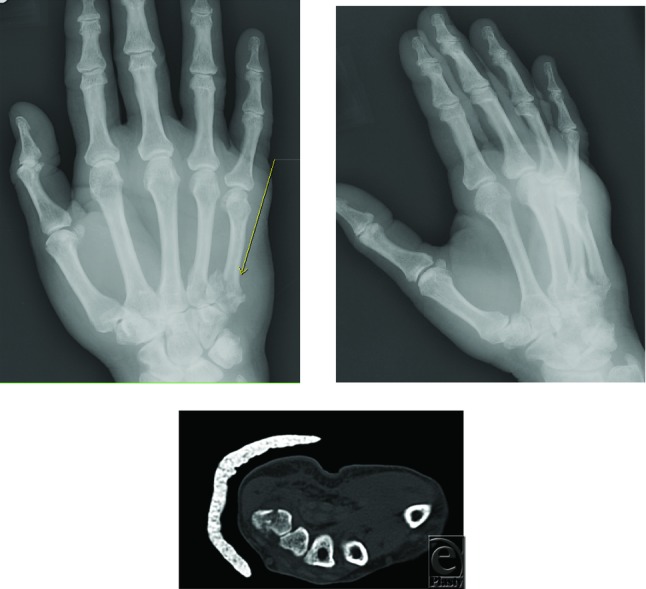


## DESCRIPTION

A 58-year-old woman was involved in a motor vehicle accident. She sustained diffuse swelling over the dorsum of her dominant hand along with a hematoma extending from the level of the metacarpophalangeal joint to the wrist. There was also extensive bruising over the palmer aspect. There was no obvious neurovascular compromise.

## QUESTIONS

1. **What is the diagnosis?**
2. **Is there any eponym for the intra-articular fractures of the base of the fifth metacarpal.**
3. **Describe briefly the mechanism causing such fracture.**
4. **What are the treatment options?**

## DISCUSSION

This is an intra-articular fracture of the fifth metacarpal.

Intra-articular fractures of the fifth metacarpal resemble Bennett's and Rolando's fractures in their pattern^1^^,^^2^ and their tendency to be unstable.^1^^,^^3^ Hence they are sometimes referred to as “Mirrored” Bennett's and Rolando's Fractures.

Striking a hard object with a closed fist was the most common cause of an intra-articular fracture of the base of the fifth metacarpal.^2^^,^^4^ Force acting on the head of the metacarpal causes a metacarpal neck fracture, but in some cases metacarpal base fracture occurs. Instability of intra-articular fractures of the fifth metacarpal base is mainly due to the strong, unopposed proximal pull of the extensor carpi ulnaris, which causes ulnar and dorsal subluxation of the main fracture fragment. The radial fragment at the base of the fifth metacarpal is held in place by the interosseous metacarpal ligament, which connects the fourth and fifth metacarpals bases.^4^

Fractures of the first metacarpal base have been well described, and reliable methods of treatment have been established. In contrast, the treatment of isolated intra-articular fractures of the base of the fifth metacarpal remains controversial. Clement^5^ first described an isolated intra-articular fracture of the base of the fifth metacarpal. Since then, multiple case reports and series have been published, but the optimal treatment of these fractures remains uncertain. The authors have recommended a variety of treatment options. Internal fixation to restore articular congruity is recommended by some authors,^1^^,^^2^ whereas others advocate cast immobilization^4^^,^^6^ or early unrestricted mobilization.^4^^,^^7^ Niechajev^2^ performed a retrospective review of 23 fractures, treated either by closed reduction and percutaneous pinning or open reduction and internal fixation. “Good” results were reported based on the subjective functional recovery, the absence of persistant tenderness, and the measurement of grip strength. Kjaer-Petersen et al^1^ analyzed a series of 64 intra-articular fractures of the base of the fifth metacarpal with special emphasis on the method of treatment and functional results. Both conservative and operative methods of treatment were used, and they reported that the alignment of 63% of the fractures was improved with open reduction and internal fixation, compared with only 20% for those treated with closed reduction and casting. These authors recommended restoration of articular congruity and internal fixation for displaced fractures. In contrast, Lundeen and Shin^4^ have shown that closed reduction and castings is an adequate and reliable treatment method for intra-articular fractures of the base of the fifth metacarpal. All fractures were reported to heal within an average of 5 weeks, and all active duty military patients returned to full duty status after an average of 6 weeks. The proponents of surgical management have shown grip weakness,^6^ longer periods of disability, and poorer outcome^7^ in patients with inadequate reduction of intra-articular fractures through the base of the fifth metacarpal. Authors recommending conservative treatment have reported the contrary.^4^

The “mirrored” Rolando's fracture of the base of the fifth metacarpal was successfully treated in this setting with open reduction and internal fixation. However, the debate continues between closed reduction and casting versus operative management. Regardless of their choice of treatment option, a surgeon's personal preference will be supported by evidence-based literature. Until a large-scale study comparing these 2 treatment options is completed, a surgeon's personal preference will continue to be acceptable standard of care.
